# Epstein-Barr Virus MicroRNA Expression Increases Aggressiveness of Solid Malignancies

**DOI:** 10.1371/journal.pone.0136058

**Published:** 2015-09-16

**Authors:** Deep Pandya, Marisa Mariani, Shiquan He, Mirko Andreoli, Manuela Spennato, Candice Dowell-Martino, Paul Fiedler, Cristiano Ferlini

**Affiliations:** Danbury Hospital Research Institute, Danbury Hospital, Danbury, Connecticut, United States of America; French National Center for Scientific Research - Institut de biologie moléculaire et cellulaire, FRANCE

## Abstract

The Cancer Genome Atlas (TCGA) microRNA (miRNA) initiative has revealed a pivotal role for miRNAs in cancer. Utilizing the TCGA raw data, we performed the first mapping of viral miRNA sequences within cancer and adjacent normal tissues. Results were integrated with TCGA RNA-seq to link the expression of viral miRNAs to the phenotype. Using clinical data and viral miRNA mapping results we also performed outcome analysis. Three lines of evidence lend credence to an active role of viral miRNAs in solid malignancies. First, expression of viral miRNA is consistently higher in cancerous compared to adjacent noncancerous tissues. Second, viral miRNA expression is associated with significantly worse clinical outcome among patients with early stage malignancy. These patients are also featured by increased expression of PD1/PD-L1, a pathway implicated in tumors escaping immune destruction. Finally, a particular cluster of EBV-miRNA (miR-BART2, miR-BART4, miR-BART5, miR-BART18, and miR-BART22) is associated with expression of cytokines known to inhibit host response to cancer. Quantification of specific viral miRNAs may help identify patients who are at risk of poor outcome. These patients may be candidates for novel therapeutic strategies incorporating antiviral agents and/or inhibitors of the PD-1/PD-L1 pathway.

## Introduction

Approximately 98% of the DNA in the human genome is unrelated to protein coding [1]. Originally thought to be “junk DNA”, non-protein encoding regions are now known to be critical to cell function. These regions contain instructions for RNA species, particularly microRNAs (miRNAs), which regulate and control transcription and translation via complex signaling networks and pathways. MiRNAs are small RNA molecules (18–22 nucleotides) with demonstrated functionality in tissue differentiation [2] and adaptation to micro-environmental stress [3, 4].

The Cancer Genome Atlas (TCGA) project begun in 2005 catalogues genetic mutations and gene and miRNA expression profiles in various cancers. Initially confined to glioblastoma multiforme, lung cancer, and serous ovarian cancer, the atlas now includes carcinomas of kidney, stomach, prostate, uterus, bladder, head & neck, thyroid, colon, rectum, and others.

TCGA project has analyzed miRNA expression in all these diseases and 85 papers have been retrieved in Pubmed at the current date using the keywords “TCGA” and “microRNA”. It is not possible in this manuscript to cite and discuss all these investigations. It is evident from such analyses that individual miRNAs play a pivotal role in conditioning the gene/protein expression pattern with some specific tissue peculiarities. However, despite some differences, these findings have confirmed the original idea that miRNAs are implicated in the pathogenesis and prognosis of cancer [5–8].

The human miRNA database (miRBase) [9] currently includes 1,881 sequences, and the list is growing. In a display of adaptive “genius”, viruses utilize miRNAs when hijacking command of infected cells. This strategy is particularly prominent among herpes viruses which account for the vast majority of the known virus-encoded miRNAs.

Many herpetic infections are long lasting and persist for the entire life of an infected patient [10]. In healthy individuals, immune control mitigates the severity of herpetic infection. Since the occurrence of cancer often requires or induces at least some immunosuppression, we suspected that herpetic viral miRNAs are expressed in cancer patients and may be involved in determining clinical features of their disease.

We postulated that multiple parallel sequencing, employed to investigate the expression of miRNAs in TCGA datasets [11], had overlooked herpetic viral miRNAs in human specimens due to the fact that only human (and not viral) miRNAs were included in the reference miRNA database. In a recently published study conducted on a large cohort of serous epithelial ovarian cancer patients, we demonstrated that this is indeed the case [12]. Herpetic miRNAs are not only relatively abundant in ovarian cancer tissues, they also drive clinical outcome. In the current analysis, we employ the same bioinformatic pipeline and methods to a broad array of solid malignancies. Our results confirm that herpes virus miRNAs are important mediators in many types of cancer and that specific miRNA species can identify subsets of patients who are at particular risk of disease progression.

## Material and Methods

### Bioinformatic analysis of TCGA data

This study is based on the analysis of TCGA data and has been deemed exempt under 45 CFR 46.101(b) by the local (BRANY) IRB, being research involving the study of existing data recorded in a manner that study subjects cannot be identified.

TCGA miRNA data are widely available as level 3 (processed/mapped) data to the research community. However, the analysis performed by TCGA consortium did not include viral miRNAs among the reference sequences utilized for mapping. In order to overcome this limitation, we downloaded the level 1 (raw/not normalized) data and performed mapping to both the human and viral miRNA genomes, as previously described with minor changes [12]. A schematic description of the pipeline is provided in S1 Fig. Briefly, Level 1 high-throughput miRNA sequencing data in. bam (compressed short sequence read alignments format) were downloaded from the TCGA data portal to the local servers and then uploaded to the CLC GW software (Qiagen). Permission to access all data was obtained from the Data Access Committee of the National Center for Biotechnology Information Genotypes and Phenotypes Database (dbGAP) at the National Institute of Health. GeneTorrent shell was installed and used to fetch data on local servers through manifest files, which were created for the study on https://browser.cghub.ucsc.edu/. All specimens sequencing data in. bam format were automatically uncompressed and separated into two sequencing files containing mapped and unmapped read alignment with reference to GRch37-lite. Both mapped and unmapped sequencing alignment files for each specimen were then extracted to obtain sequencing reads. Quality and quantity control was performed to generate reports for each specimen, including mapped and unmapped total number of reads and average read length. All the data with extracted sequencing reads from each specimen were further analyzed through small RNA Sequencing analysis module. They were first, trimmed and filtered with maximum read length of 55bp and minimum read length of 15bp. As a reference for small RNA sequencing reads mapping, both human and viral mature miRNA database were downloaded and used as references. Mapping results were then classified into human miRNAs and viral miRNAs. Viral miRNAs from EBV (Epstein Barr Virus), HSV-1 (Herpes Simplex Virus 1), HSV-2 (Herpes Simplex Virus 2), KSHV (Kaposi Sarcoma Herpes Virus) and CMV (Cytomegalovirus), were selected. One of the risks in a similar project is that some reads could map within non-miRNA loci within the human genome. At variance of the previously published study in ovarian cancer [12], we removed from the analysis four viral miRNAs (miR-H15 and miR-H17 from HSV-1 and miR-H10 and miR-H24 from HSV-2) whose expression was detected in >98% of the specimens. Mapping was performed for each specimen through “annotate and merge” tool of the CLC software. As anticipated, a significant number of mapped reads to mature viral miRNAs was noticed in the reads originally unmapped to the human genome. However, reads mapping to viral miRNAs were obtained also in the reads originally mapped to the human genome, even if priority was always kept for the mapping toward GRch37-lite. Each read was allowed to map only once either into the human or viral miRNA database. All mapped miRNAs per specimen were normalized to TPM (transcript per million reads) values with total number of specimen sequencing reads, as previously described [12]. Clinical data were then integrated with specimen sequencing analysis data in order to perform outcome analysis. Analysis was performed in two datasets. The first included all patients (n = 600) available in which at least one sample from both noncancerous (biospecimens n = 1,199) and cancer (specimens = 1,184) were sequenced (Tab. 1). The second dataset included only sample from cancer tissues (biospecimens n = 3,395, Tab. 2). The second dataset was enriched for malignancies showing the highest expression of viral miRNAs. Level 3 RNA expression (RNA-seq) data were also retrieved from TCGA portal.

### Ethics statement

All the samples here analyzed were recruited as a part of the NIH sponsored TCGA project and are available through the TCGA data portal https://tcga-data.nci.nih.gov. All the participants of the study gave written consent. Authors of this study got access to the TCGA data which are fully de-anonymized. Local investigators never interacted with the TCGA patients. For this reason, this analysis has been deemed exempt by the Danbury Hospital/BRANY IRB under CFR 46.101(4) exemption.

### Statistical Analysis

Expression data served to cluster the correlation across the mapped miRNAs in an unsupervised way, with the use of the Spearman test R values and cluster on correlation function of the JMP11 software. The significance of increased or decreased expression of viral miRNAs along with other factors was computed with the use of t-test or Fisher exact test of numerical or categorical variables. For categorization of numerical variables related to viral miRNA expression, negative and positive were defined as 0 and >0 TPM, respectively. Outcome analysis was performed using overall survival in months in a multivariate Cox model inclusive of age and stage. For Kaplan-Meier analysis, Wilcoxon test was employed to determine whether the difference in survival was statistically significant across categorical groups. We used Spearman analysis and strict Bonferroni correction to identify correlation among individual miRNAs of interest. For pathway analysis, we relied on the Panther classification system [13] and the classification tools available at http://www.pantherdb.org/. Type I error for all the tests was set at 5% and all the analyses were performed with the JMP11 software package.

## Results

**Fig 1:**
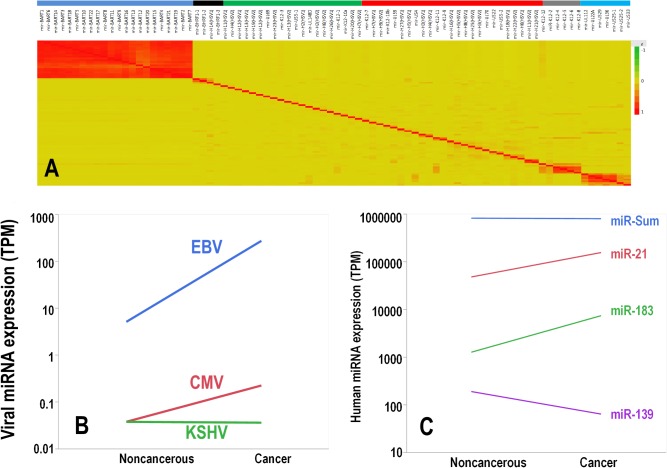
A: Heat map of the cluster on correlation among the mapped viral miRNAs. Yellow background means no correlation, while the red intensity signal is proportional to the R value of the Spearman correlation. No inverse correlation was noticed. Six independent clusters (color bar on the top of the figure) have been obtained through unsupervised clustering. The first cluster (blue line) includes all the 22 EBV-miRNA encoded by the BART gene; the second cluster (black line) includes all the three EBV-miRNA encoded by the BHRF gene (and miR-H14 from HSV-1). The third cluster (green line) comprises 9 HSV-2, 5 HSV-1, 3-CMV and 3-KSHV viral miRNAs. The fourth (red line) includes 9 HSV-1, 7 HSV-2, 4 KSHV and 5 CMV viral miRNAs. The fifth (gray line) and sixth are pure clusters (light blue line) comprising 6 and 7 viral miRNAs encoded by KSHV and CMV, respectively. B: Line chart showing average expression of viral miRNA in noncancerous vs. cancer tissues separated by viral species. Differences were statistically significant (paired t test, p<0.001) for all viruses except KSHV. C: Line chart showing average expression of miR-21, miR-183, miR-139 and the overall sum of all human miRNA miR-Sum. Differences were statistically significant (paired t test, p<0.001) for all except miR-Sum.

This study was aimed at analyzing expression of viral miRNA from EBV, HSV-1, HSV-2, KSHV and CMV in cancer patients. Details of the sequencing pipeline are provided in the material and methods and in the supporting information. In sequencing projects there is always the risk of mismapping sequences which have homology “on” (other miRNAs) or “off” (other non-miRNA loci) target. One of the advantages of mapping viral genomes within human samples is that in a correct mapping situation you could expect that several viral miRNAs, encoded by the same viral gene, are co-expressed. For this reason, as first quality control of mapping, we performed cluster analysis on pairwise Spearman correlation of all the mapped viral miRNAs. Results strongly support a meaningful mapping (Fig 1A). If divided in six independent unsupervised clusters, EBV expression was confined almost purely in two clusters, with the EBV-miRNA encoded in the BART and BHRF viral genes all co-expressed in two independent clusters. A strong tendency to co-expression was noted also for seven and six viral miRNAs encoded by CMV and KSHV, respectively. No evident clusters of significant co-expression were noticed instead in the HSV-1 and HSV-2 viruses. These findings are in good keeping with recent reports indicating that some viral miRNAs encoded by HSV-1 (and probably by HSV-2) are not actually expressed [14]. For these reasons we considered for further analyses only the viral miRNA species mapped in EBV, CMV and KSHV.

In the 600 patients (Table 1) with solid malignancies where both noncancerous and cancer tissues were available for comparison, we successfully mapped 21.3 billion sequences. For most of the patients at least two samples (biospecimens) were available with few exceptions (Table 1). We detected a significant number of viral miRNAs in both tissue types with greater than 96% derived from EBV. On average, 12.2 TPM viral miRNAs were mapped in cancer tissues, more than double the average of 5.5 TPM in adjacent noncancerous tissues. Paired t-test by viral type demonstrated that all virus-specific miRNAs (with the exception of KSHV) where increased in cancer vs. noncancerous tissues (Fig 1B). This difference cannot be attributed to an increased production of miRNA in cancer tissues. In fact, the sum of all the 1,881 human miRNAs included in the mapping exhibited a not significant (p>0.05) trend toward a decreased miRNA expression in the cancer tissue (Fig 1C). Separation of normal and cancer tissues across the samples seems validated by human miRNA analysis. Human miRNAs typically overexpressed in cancer tissues such as miR-183 and miR-21 were significantly overexpressed in the malignancies (p<0.001, Fig 1B), while the human miRNA with the highest overexpression in the noncancerous tissue was miR-139 (p<0.001, Fig 1C).

**Table 1:** Number of paired Cancer/normal tissues mapped for viral miRNA expression.

Disease	Normal	Tumor	All	Patients
Bladder Carcinoma (BLCA)	35	35	70	19
Breast Cancer (BRCA)	206	213	419	102
Head & Neck Squamous Carcinoma (HNSC)	81	81	162	42
Chromophobe Renal Cell Carcinoma (KICH)	50	50	100	25
Renal Clear Cell Carcinoma (KIRC)	142	143	285	71
Renal Papillary Cell Carcinoma (KIRP)	60	60	120	34
Liver Hepatocellular Carcinoma (LIHC)	96	96	192	48
Lung Adenocarcinoma (LUAD)	92	92	184	46
Lung Squamous Cell Carcinoma (LUSC)	90	90	180	45
Prostate Adenocarcinoma (PRAD)	100	100	200	50
Stomach Adenocarcinoma (STAD)	67	65	132	38
Papillary Thyroid Carcinoma (THCA)	118	118	236	59
Uterine Corpus Endometrial Carcinoma (UCEC)	62	41	103	21
**Disease = All**	**1,199**	**1,184**	**2,383**	**600**

**Fig 2:**
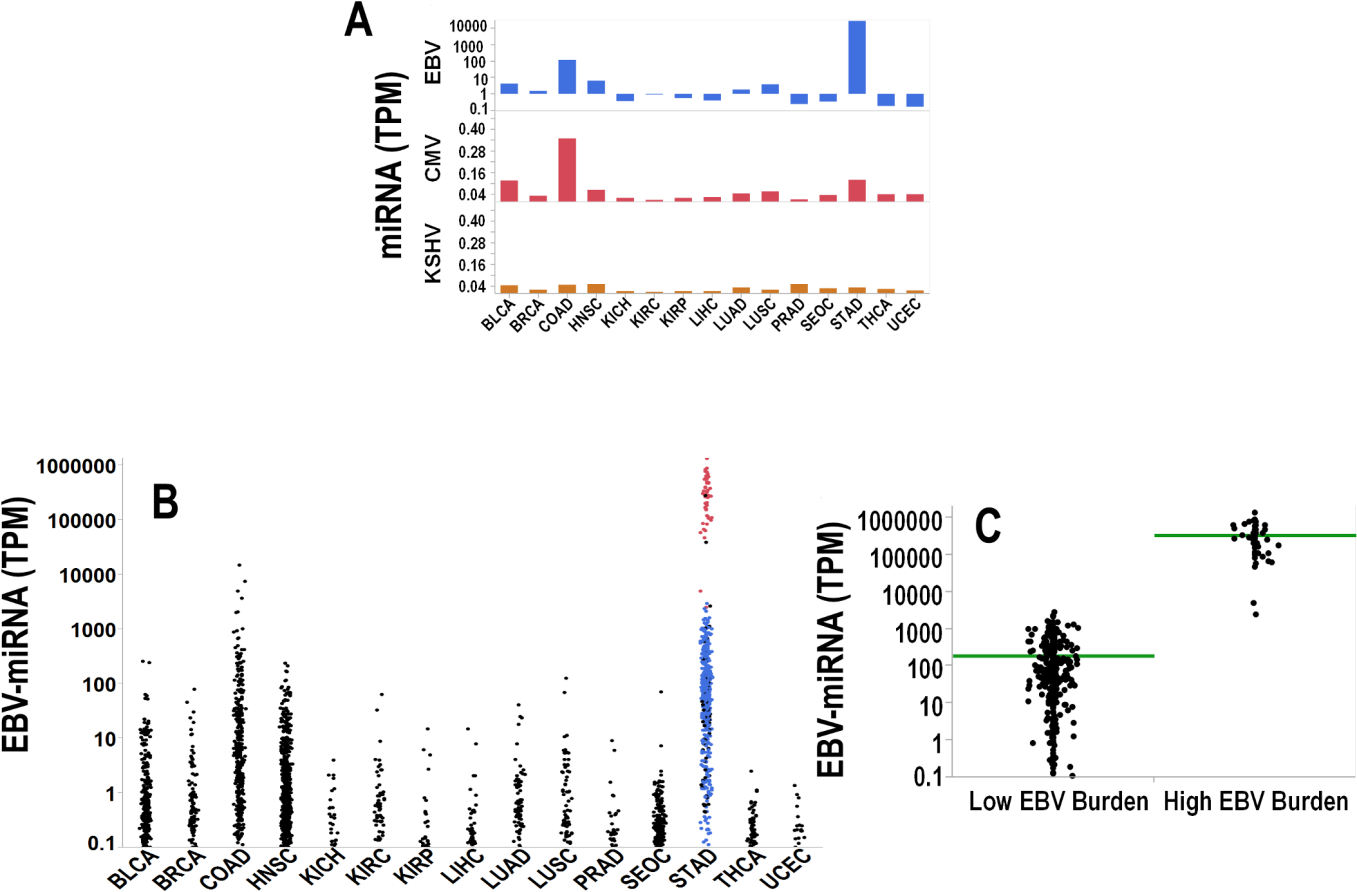
A: Bar charts showing the average viral miRNA expression in cancer tissues grouped by virus species. From top to bottom: EBV, CMV and KSHV. Legends of the x-axis are taken from TCGA nomenclature and are as follows: Bladder Carcinoma (BLCA), Breast Cancer (BRCA), Colon Adenocarcinoma (COAD), Head & Neck Squamous Carcinoma (HNSC), Chromophobe Renal Cell Carcinoma (KICH), Renal Clear Cell Carcinoma (KIRC), Renal Papillary Cell Carcinoma (KIRP), Liver Hepatocellular Carcinoma (LIHC), Lung Adenocarcinoma (LUAD), Lung Squamous Cell Carcinoma (LUSC), Prostate Adenocarcinoma (PRAD), Serous Ovarian Cancer (SEOC), Stomach Adenocarcinoma (STAD), Papillary Thyroid Carcinoma (THCA), and Uterine Corpus Endometrial Carcinoma (UCEC). B: Dot plots depicting the expression of EBV-miRNAs across all the tumor types as labeled in A. In color the samples overlapping with the TCGA study [15] are depicted. Samples in blue and red are classified in [15] as low and high EBV burden, respectively. In C the difference in the expression for samples at high/low EBV burden is significant (p<0.001).

In a second analysis of TCGA data using the same pipeline and approach, we did not restrict cohort inclusion to only those patients with paired noncancerous and cancer tissues. Rather, we captured a larger cohort of 2,330 patients affected by solid malignancies (Table 2). Overall, we mapped additional 32 billion sequences and found a much higher average viral miRNA load as compared to the first cohort (4,612 TPM vs. 12.2 TPM). This dramatic difference relates to the fact that we enrolled in the second cohort additional 1,760 patients including cancer types characterized by high viral miRNA expression (stomach (STAD), colon (COAD), bladder (BLCA) and Head & Neck (HNSC)). The average expression of viral miRNA grouped by cancer type and virus species is shown in Fig 2A. The cumulative expression of EBV-miRNA is depicted in Fig 2B. Among the available cancer tissues, a previous TCGA report identified in the stomach adenocarcinoma a subset of 26 patients with high EBV burden tumors [15]. In keeping with this analysis, our analysis of viral miRNA expression revealed that in the same set of 26 patients (48 biospecimens) we detected the highest expression of EBV-viral miRNAs (Fig 2B&2C). This high level of expression was never detected in the other malignancies and most likely is related to a direct EBV-infection occurring in the gastric cancer cells, as previously suggested [15].

**Table 2:** Number mapped for viral miRNA expression in cancer patients.

Disease	Number	Difference*
Bladder Carcinoma (BLCA)	217	198
Breast Cancer (BRCA)	106	4
Colon Adenocarcinoma (COAD)	402	402
Head & Neck Squamous Carcinoma (HNSC)	420	408
Chromophobe Renal Cell Carcinoma (KICH)	25	0
Renal Clear Cell Carcinoma (KIRC)	71	0
Renal Papillary Cell Carcinoma (KIRP)	31	-3
Liver Hepatocellular Carcinoma (LIHC)	45	-3
Lung Adenocarcinoma (LUAD)	46	0
Lung Squamous Cell Carcinoma (LUSC)	38	-7
Prostate Adenocarcinoma (PRAD)	50	0
Serous Ovarian Cancer (SEOC)	465	465
Stomach Adenocarcinoma (STAD)	336	298
Papillary Thyroid Carcinoma (THCA)	57	-2
Uterine Corpus Endometrial Carcinoma (UCEC)	21	0
**Disease = All**	**2,330**	**1,760**

*As compared with the dataset noncancerous vs. cancerous tissue reported in Tab. 1

**Fig 3:**
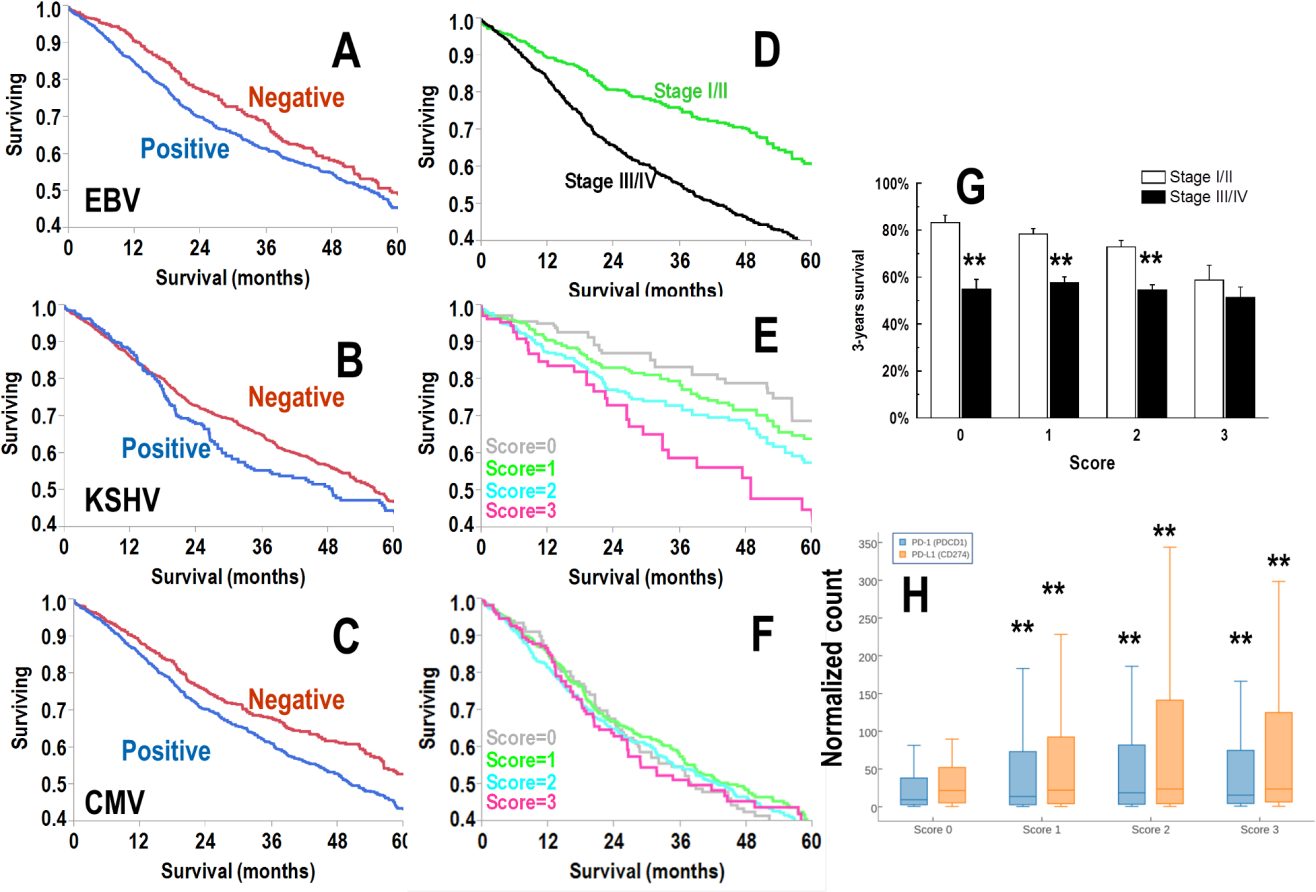
A-C: Kaplan-Meier analysis for the expression of viral miRNA expression (A, B and C for EBV, KSHV and CMV, respectively). Patients were categorized as positive or negative if the TPM sum was >0 or 0, respectively. D: Kaplan-Meier analysis according to tumor stage. In green and black are AJCC stage I/II and stage III/IV, respectively (p<0.001). E&F: Kaplan-Meier analysis according to a cumulative score for no expression (score 0) or expression of one, two or three viruses such as EBV, KSHV and HSV-2. Score 3 indicates the expression of viral miRNA from all the three viruses. In gray, green, light blue and magenta are scores 0, 1, 2 and 3, respectively. In E, the chart refers to early AJCC stage (I/II), while F shows late AJCC stage (stage III/IV). Differences across the four groups is significant (p<0.001) only in E. G: Bar chart showing the three years survival in patients according to the viral score. Bar and error bars are probability of survival at 3-years and its standard deviation, respectively. Double asterisks indicate a significant difference (t-test, p<0.05). Early diagnosis is not protective with score 3 patients exhibiting not significant differences in terms of 3-years survival H: Box-whisker plot showing the expression of PD-1 (*PDCD1*) and PD-L1 (*CD274*) according to viral score Box and whisker represent the 25^th^-75^th^ percentile range and the 5^th^-95^th^ percentile range, respectively. Horizontal line in the plot is the median. Double asterisks indicate a significant difference as compared with score 0 (Tukey test, p<0.001).

To perform outcome analysis at the viral species level, patients were categorized as positive if the sum of pooled viral miRNA expression by species was >0 TPM or negative if the expression was equal to 0 TPM. We found the following average positivity rates across cancer types: EBV 76%, CMV 67%, and KSHV 18%. EBV and KSHV positive patients showed significantly (p<0.05) worse overall survival (OS) in a Kaplan-Meier model (Fig 3A–3C). Given this finding, we created a viral score wherein one point each was assigned for EBV, CMV and KSHV positivity. As examples, a patient with no expression of these viral miRNAs scored 0, whereas a patient expressing all three viral miRNAs scored 3. Classifying patients by viral score yielded 13% with score 0; 38% with score 1; 38% with score 2 and 11% with score 3. We also grouped patients according to early disease (AJCC stages I/II, 42%) and advanced disease (AJCC stages III/IV, 58%). As expected, AJCC stage was a potent prognosticator (p<0.001) in Kaplan-Meier analysis (Fig 3D). Viral score was also a powerful predictor of outcome, but only in early disease (p<0.001, Fig 3E) and not advanced disease (p>0.05, Fig 3F). Three-year survival of patients with score 3 did not show any significant difference between patients diagnosed early and late stage (Fig 3G).

One of the advantages of the TCGA dataset consists in the availability of RNA-seq data along with the miRNA seq. This makes possible to connect the viral miRNA scoring with the gene expression profile. Since it has been recently reported that herpetic viral reactivation is associated with stimulation of the PD-1/PD-L1 pathway [15]- a well-known inducer of anticancer immune suppression-, we analyzed the expression of PD-1/PD-L1 according to viral scoring (Fig 3H). Patients with score 0 expressed levels of PD-1 and PD-L1 significantly (p<0.001) lower than those noticed in patients with score 1–3, thereby confirming in a large set of patients that the endogenous reactivation of latent herpetic infections is associated with PD-1/PD-L1 activation.

Since in this study we performed outcome analysis in multiple malignancies with different degree of aggressiveness, it was important to exclude that the noticed effects were dependent on sampling problems. To test this possibility, in the AJCC stages I/II patients we generated randomly four groups 10,000 times, assigning to each group the same number of patients we had in each of the 4 viral scores. For each of the 10,000 grouping we performed again Kaplan-Meier analysis and none of the random grouping combinations showed statistically significant difference between all the pairwise groups as we obtained from our original analysis. These findings demonstrate that the differences we observed across the viral score groups are not dependent on random grouping effects.

**Fig 4:**
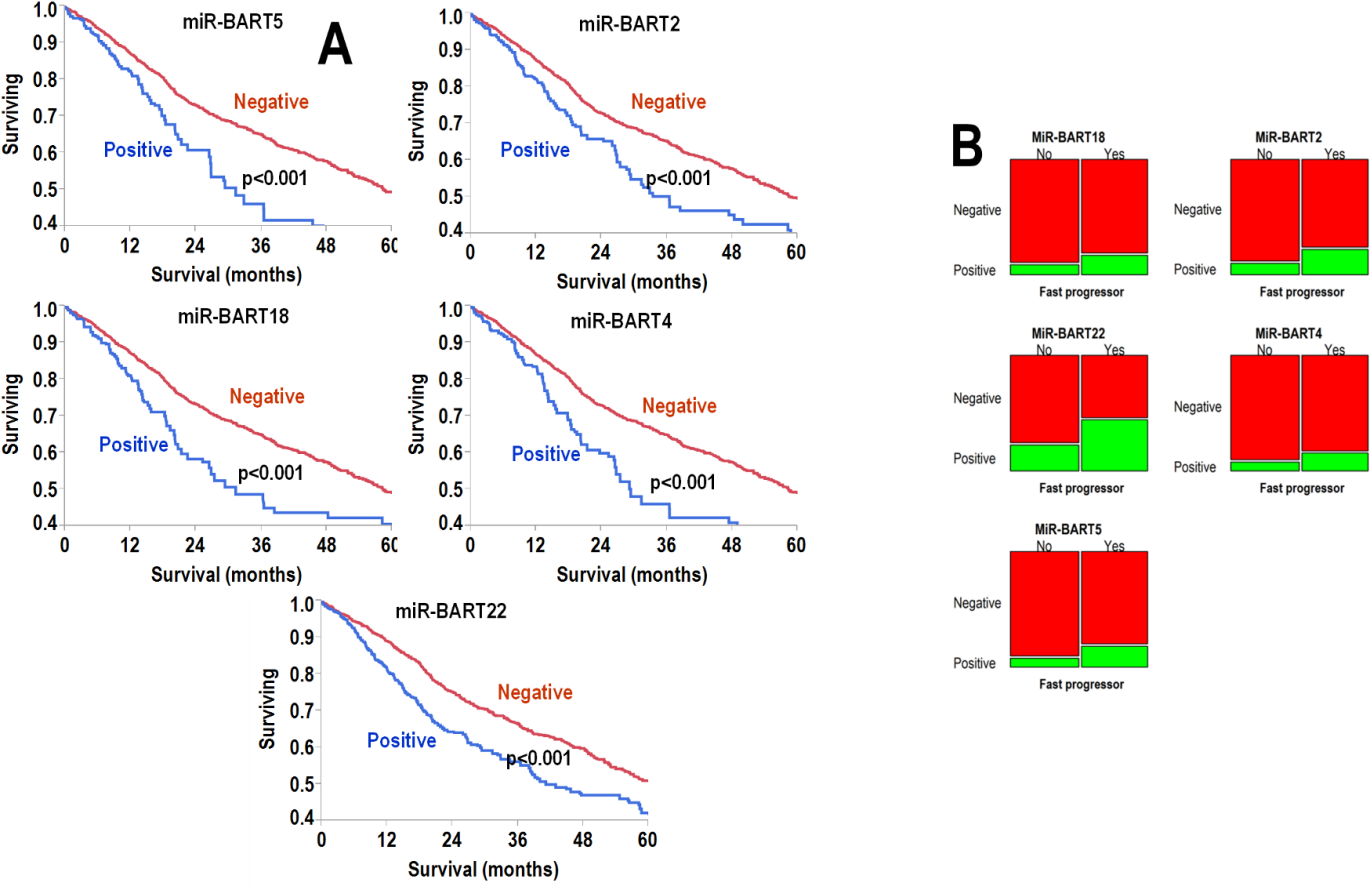
A: Kaplan-Meier analyses for the expression of the viral miRNA significant in multivariate Cox analyses. In the top panel from left to right: MiR-BART5, MiR-H3 and MiR-BART2. In the bottom panel from left to right: MiR-BART18, MiR-BART4 and MiR-BART22. Expression of viral miRNA was correlated with poor outcome in all cases. Differences are highly significant (Wilcoxon test, p<0.001). B: Mosaic plots showing the percentage of patients categorized for the expression of each significant viral miRNA. On the y-axes, patients were classified as negative (red pattern) or positive (green pattern) for each viral miRNA. On the x-axes, patients were classified as fast progressors (yes) or not (no). By definition, fast progressors succumbed to disease within 18 months from diagnosis. Left column top to bottom: MiR-BART18, MiR-BART22 and MiR-BART5; right column top to bottom: MiR-BART2, MiR-BART4 and MiR-H3. Fisher exact test demonstrates that the increased frequency of fast progression in all the patients positive for the EBV-viral miRNA was significant (p<0.001).

Next, we analyzed outcome according to individual rather than pooled species expression of viral miRNAs. Here also, patients were categorized as positive or negative if the expression of a given viral miRNA was >0 or 0 TPM, respectively. The results in each disease are summarized in S1 Table. We assessed the effect of positivity vs. negativity on survival in Kaplan-Meier analysis, univariate Cox model, and multivariate Cox model including age and stage, which were both statistically significant predictor in univariate analysis (S2 Table). After strict Bonferroni correction, six viral miRNAs (miR-BART cluster comprised of miR-BART2, miR-BART4, miR-BART5, miR-BART18, and miR-BART22 from EBV) were significant predictors of poor outcome; Kaplan-Meier curves for each of these miRNAs are shown in Fig 4A. Fisher exact test of the EBV miR-BART cluster miRNAs also showed significant increases in positivity status among patients with fast progression, defined as death within 18 months from diagnosis (Fig 4B).

**Fig 5:**
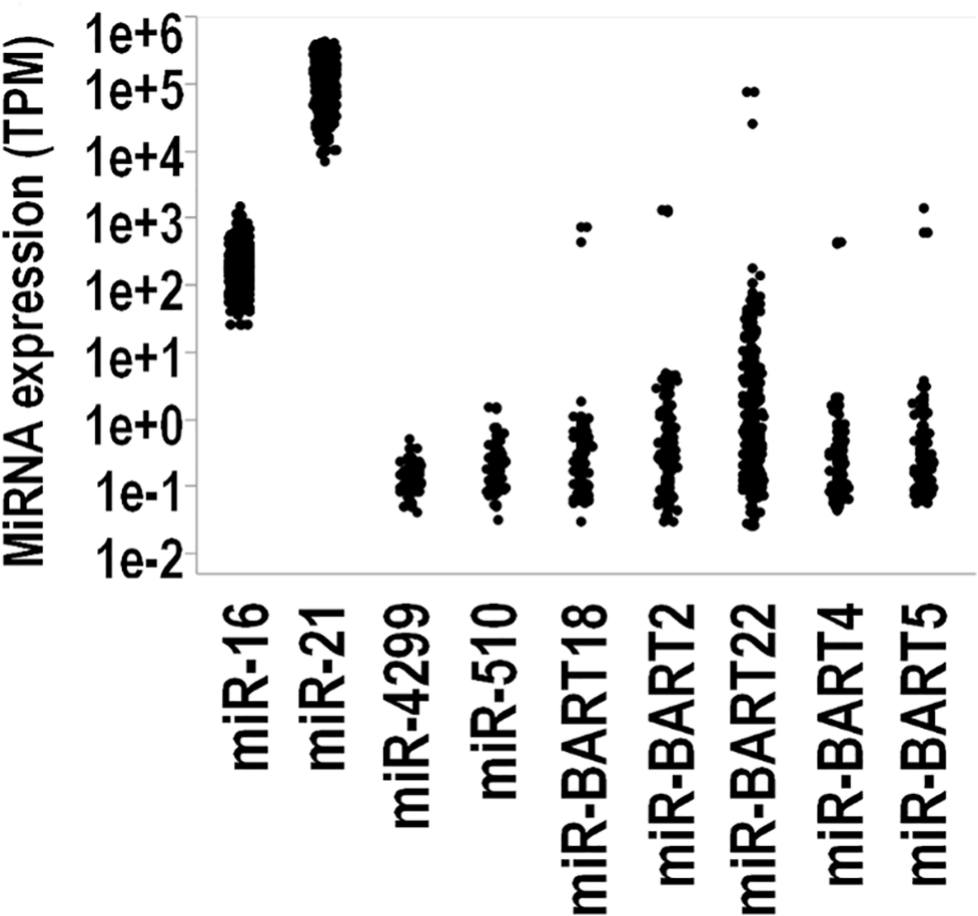
Dot chart showing the viral miRNA expression of a panel of human and viral miRNAs. From left to right MiR-16, MiR-21, MiR-4299, MiR-510, MiR-BART18, MiR-BART2, MiR-BART22, MiR-BART4, MiR-BART5 and MiR-H3. Each dot corresponds to the expression measured in a sample. Expression levels of viral miRNAs overlap with those of miR-510 and MiR-4299.

In keeping with the analysis presented in Fig 1A, expression levels of the EBV miR-BART cluster miRNAs were highly correlated with each other (range min-max ρ = 0.89–0.99, p<0.0001). Relative to human miRNAs, these six viral miRNAs were expressed at average levels below miR-16 and miR-21 and similar to miR-510 and miR-4299 (Fig 5).

**Fig 6:**
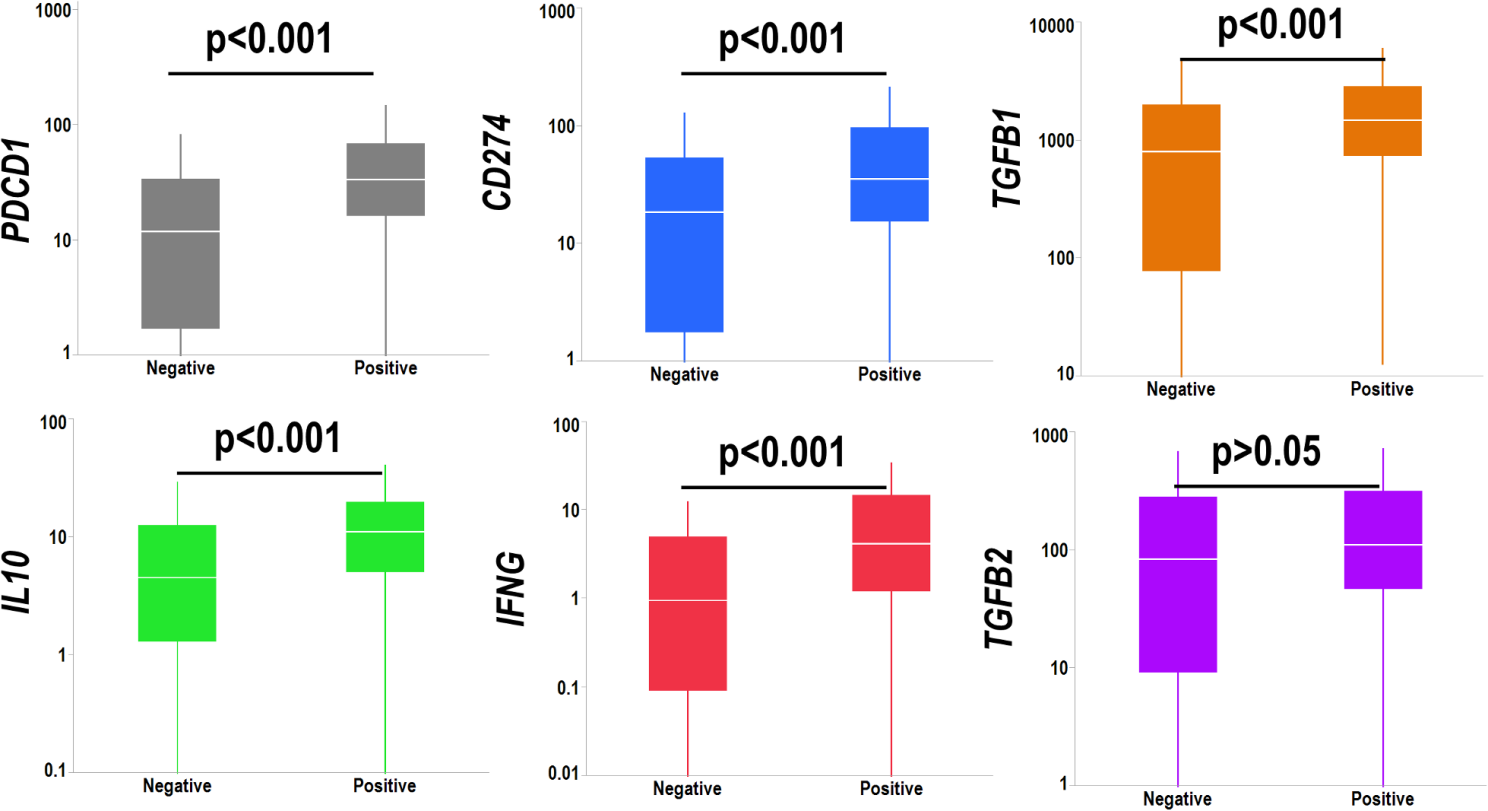
Box-whisker plot showing the transcript levels of *IL10* (gray chart), *TGFB1* (red chart), *IL2* (blue chart) and *TGFB2* (green chart) according to the expression of the EBV-miRNA cluster. Vertical lines, box and horizontal white line correspond to min-max range, 25–75^th^ percentile range and median, respectively. Cancer patients positive for the EBV-miRNA cluster showed significant (p<0.05) overexpression (t-test) of *IL10* and *TGFB1*, but not *IL2* and *TGFB2*. Data are reported as normalized count as provided in the TCGA level 3 data.

Since miRNAs regulate transcription of target genes, we searched for genes whose expression levels correlated (after strict Bonferroni correction) with the EBV miR-BART cluster miRNAs. MiRNAs are generally suppressive, and the negatively correlated genes are reported according to gene functional groups in S2 Fig. The EBV miR-BART cluster exhibited a relative abundance of suppressed genes belonging to metabolism and cell adhesion pathways. We also analyzed a panel of factors involved in the regulation of anticancer immunity such as PD-1 (*PDCD1*), PD-L1 (*CD274*) TGFβ1 (*TGFB1*), IL-10 (*IL10*), IFNγ (*IFNG1*) and TGFβ2 (*TGFB2*). EBV miR-BART cluster was associated with a significant upregulation of all these factors with the exception of TGF-β2 (Fig 6).

## Discussion

We recently reported the presence and activity of viral miRNAs in serous ovarian carcinoma [12]. The current study extends this work and represents the first comprehensive clinical investigation of viral miRNA expression in a wide array of solid malignancies. Utilizing miRNA-seq level 1 data from TCGA consortium, we mapped the expression of tumor miRNAs to known viral genomes in a panel of human cancers. We show that increased viral miRNA expression in epithelial malignancies compared to noncancerous tissues is a common phenomenon across many tumor types. EBV is foremost among the viruses adept at miRNA synthesis in human cancers. A ubiquitous human herpes virus, EBV has a previously well-documented oncogenic role in solid malignancies such as nasopharyngeal cancer and stomach adenocarcinoma [16]. TCGA dataset does not include nasopharyngeal carcinoma for comparison leaving stomach adenocarcinoma as the disease with the highest EBV miRNA expression in our analysis.

Three pathogenetic models can explain the expression of viral miRNAs in solid malignancies [12]: i) direct infection of epithelial cancer cells with production of viral miRNAs within these cells; this mechanism seems operating in a subset of 26 stomach adenocarcinoma patients expressing very high levels of EBV-miRNAs; ii) local production in tumors by non-neoplastic inflammatory or stromal cells (for example, B lymphocytes in EBV related tumors); iii) viral miRNA production in remote sites of infection with release of viral miRNAs into the circulation followed by tumor uptake. Irrespective of which model is most accurate, patients with early stage cancer (AJCC Stage I/II) and an absence of expression of viral miRNAs have the most favorable clinical outcome, while early stage patients with multiple viral miRNAs have relatively poor survival. Presence, absence or number of viral miRNAs does not appear to alter survival of patients diagnosed at late stages (AJCC Stage III/IV), thus suggesting that the impact of viral miRNA expression is maximal in those patients with the highest probability of responding to treatment.

In aggressive cancers anticancer immunity is suppressed via multiple mechanisms, including PD-1 and PD-L1 activation [17]. The immune response against cancer and endogenous herpes viruses share common pathways [18]. In this sense, re-activation of latent herpetic infections can enhance the inhibition of the anticancer immunity, thus decreasing the efficacy of anticancer therapies, even if diagnosis is made at early stage. In this context, viral miRNAs may exert their deleterious activity via induction of cytokines, including IL-10 and TGF-β1, which suppress the immune response during virus induced carcinogenesis [19–22] and counteract the antiviral activity of IFNγ [23]. Indeed, we document in this study that the expression of the EBV miR-BART cluster (miR-BART2, miR-BART4, miR-BART5, miR-BART18 and miR-BART22) present in 37% of patients is associated with a significant and specific up-regulation of PD-1, PD-L1, IL-10 and TGF-β1. Immune suppression not only favors tumor growth but also enhances viral replication in a positive feedback loop. The EBV miR-BART cluster is a likely trigger of this loop since it correlates with an aggressive cancer phenotype in univariate and multivariate analysis. It also shows a two-fold prevalence increase in treatment refractory patients demonstrating rapid clinical progression.

The EBV-miRNA cluster negatively correlates most often with genes regulating the activation of metabolism and cell adhesion pathways and likely promotes cancer cell resistance to hypoxia and poor nutrient supply along with invasiveness. Prior reports of EBV-infected cells showed similar results [24–26].

The strength of the investigation is coming from the high quality of the TCGA data, the number of patients investigated and the quality of mapping we produced, particularly within the EBV-miRNA which are the most expressed species across the investigated malignancies. The weakness of the study is that the correlation between miRNA expression and possible targets has been not validated and despite the use of strict Bonferroni correction some correlations may be indirect or simply obtained by chance. Moreover, even if not due to randomness, correlations may be indirect and at the current stage of the investigation we do not know if miRNA are simply a biomarker of viral load or instead active mediator of the suppression of the anticancer immunity.

Despite these limitations, this study demonstrates the presence of viral miRNAs in a diverse array of solid malignancies wherein they behave as prognostic biomarkers. Particularly relevant in patients with early stage cancer, viral miRNAs may serve to identify patients with a suppressed anticancer immunity, which may lead to treatment failure and rapid tumor progression even if cancer is diagnosed at early stage. These patients may be candidates for PD-1/PD-L1 targeted treatments and/or novel therapies specifically designed to disrupt virus-mediated cytokine production, with the intent to elicit both anticancer and antiviral response.

## Supporting Information

S1 Fig Schematic description of the bioinformatic pipeline used to map viral miRNA in the TCGA miRNA-seq dataset. (DOCX)

S2 Fig Pie charts showing the functional pathways negatively correlated with the EBV miRNA cluster (MiR-BART18, MiR-BART2, MiR-BART22, MiR-BART4, MiR-BART5, n = 174). The most commonly represented functional pathways were Metabolism and Cell Adhesion for the EBV-miRNA.
(DOCX)

S1 Table Expression of the individual viral miRNA across all the diseases. (XLSX)

S2 Table Outcome analysis of the individual 52 viral miRNA quantified in the TCGA dataset. (XLS)
