# Supplementary material for: Epstein-Barr Virus MicroRNA Expression Increases Aggressiveness of Solid Malignancies
Source: PLoS One. 2015 Sep 16;10(9):e0136058. doi: 10.1371/journal.pone.0136058 (PMC4573609; doi:10.1371/journal.pone.0136058)
Supplement: S1 Fig — (DOCX) [file pone.0136058.s001.docx]

**Supplementary Fig. S1**

Schematic description of the bioinformatic pipeline used to map viral miRNA in the TCGA miRNA-seq dataset

**
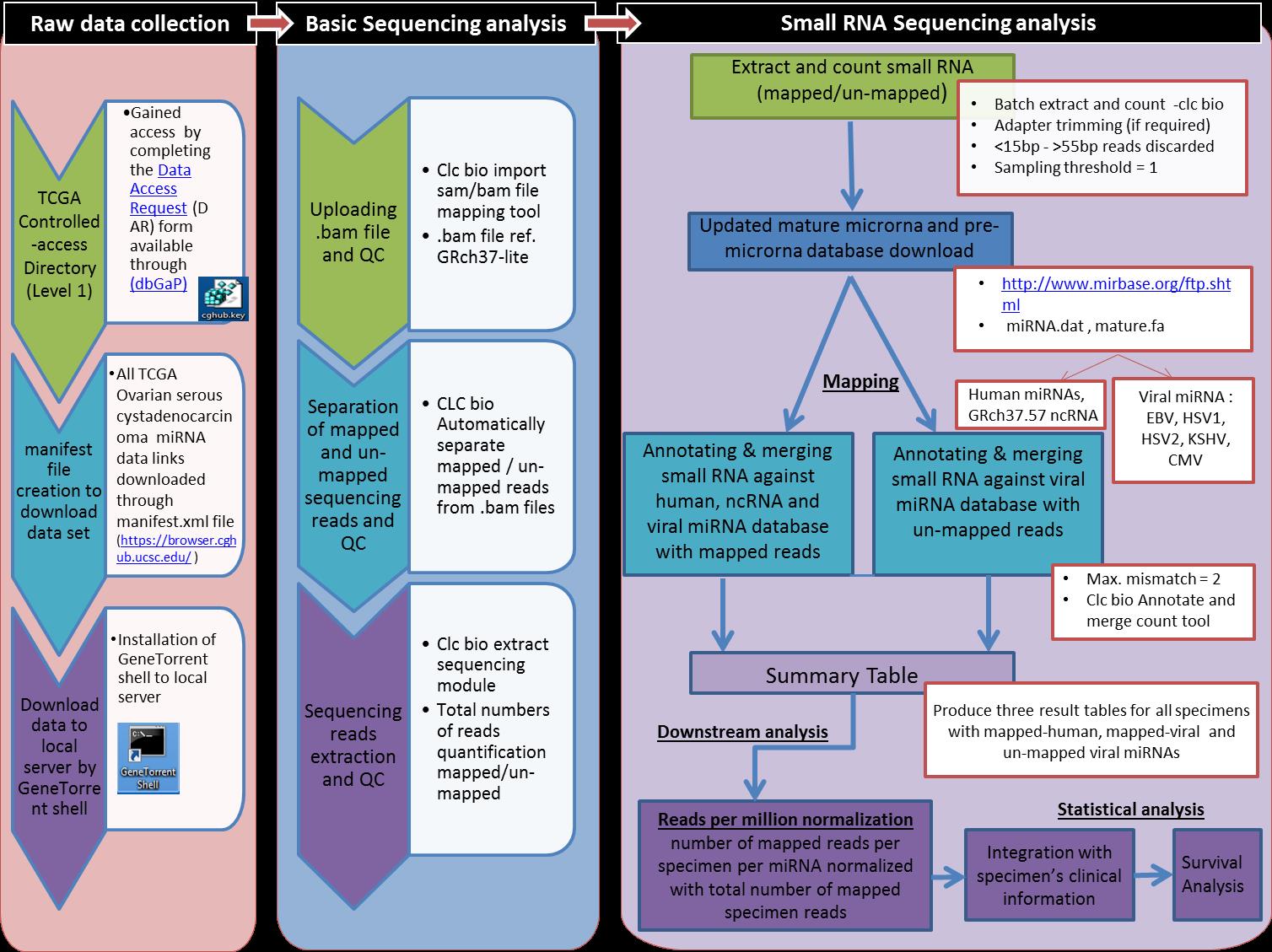
**
