# Supplementary material for: Epstein-Barr Virus MicroRNA Expression Increases Aggressiveness of Solid Malignancies
Source: PLoS One. 2015 Sep 16;10(9):e0136058. doi: 10.1371/journal.pone.0136058 (PMC4573609; doi:10.1371/journal.pone.0136058)
Supplement: S2 Fig — The most commonly represented functional pathways were Metabolism and Cell Adhesion for the EBV-miRNA. (DOCX) [file pone.0136058.s002.docx]

**Supplementary Fig. S2** Pie charts showing the functional pathways negatively correlated with the EBV miRNA cluster (MiR-BART18, MiR-BART2, MiR-BART22, MiR-BART4, MiR-BART5, n=174). The most commonly represented functional pathways were Metabolism and Cell Adhesion for the EBV-miRNA cluster.
